# A commentary on the implications of medication prescription rights for the chiropractic profession

**DOI:** 10.1186/s12998-016-0114-y

**Published:** 2016-08-24

**Authors:** Peter C. Emary, Taco A. W. Houweling, Martin Wangler, Stephen J. Burnie, Katherine J. Hood, W. Mark Erwin

**Affiliations:** 1 201C Preston Parkway, Cambridge, ON N3H 5E8 Canada; 2 Department of Chiropractic Medicine, University Hospital Balgrist, Forchstrasse 340, 8008 Zürich, Switzerland; 3 Medizinisches Zentrum KurWerk, Poststrasse 9, CH 3400 Burgdorf, Switzerland; 4 Canadian Memorial Chiropractic College, 6100 Leslie Street, Toronto, ON M2H 3J1 Canada; 5 11 King Street West, Suite C-120, Toronto, ON M5H 4C7 Canada; 6 Department of Surgery, Divisions of Neurological and Orthopaedic Surgery, University of Toronto, Toronto Western Hospital, 399 Bathurst Street, KD 5-407, Toronto, ON M5T 2S8 Canada; 7 Krembil Research Institute, 60 Leonard Street, Toronto, ON M5T 2S8 Canada

**Keywords:** Drug prescriptions, Chiropractic, Knowledge, Attitudes, Behaviour, Evidence-based practice

## Abstract

There is a growing desire within the chiropractic profession to expand the scope of practice to include limited medication prescription rights for the treatment of spine-related and other musculoskeletal conditions. Such prescribing rights have been successfully incorporated into a number of chiropractic jurisdictions worldwide. If limited to a musculoskeletal scope, medication prescription rights have the potential to change the present role of chiropractors within the healthcare system by paving the way for practitioners to become comprehensive specialists in the conservative management of spine / musculoskeletal disorders. However, if the chiropractic profession wishes to lobby to expand the scope of practice to include limited prescriptive authority, several issues must first be addressed. These would include changes to chiropractic education and legislation, as well as consideration of how such privileges could impact the chiropractic profession on a more theoretical basis. In this commentary, we examine the arguments in favour of and against limited medication prescription rights for chiropractors and discuss the implications of such privileges for the profession.

## Background

Despite a growing number of surveys demonstrating a positive attitude among chiropractors and patients towards the limited use of medication within chiropractic practice, chiropractors remain unable to prescribe medication in most parts of the world [1–8] (2007 and 2011 Ontario Chiropractic Association member surveys: B. Haig; personal communication, 3 November 2014). In many countries, chiropractors also lack direct access to musculoskeletal (MSK) diagnostic imaging and laboratory testing – limitations that have real implications for the clinician in accurately diagnosing and managing their patient. Meanwhile, allied health care professions such as optometry, chiropody, and naturopathy have been steadily expanding their respective scopes’ of practice and gaining limited medication prescription rights relevant to their areas of training and expertise [9–11]. Most notable for chiropractors is that physiotherapists are also interested in and have been granted prescriptive authority in some countries [12, 13]. Moreover, there are increasing examples of physiotherapists with advanced training in medication prescription and diagnostic testing who now manage patients with MSK disorders at the primary care level [14–16].

Currently within the chiropractic profession, three jurisdictions have incorporated the prescription of limited medications into their scope of practice. These include Switzerland [1], New Mexico (USA) [17], and most recently, Liechtenstein (C. Mikus; personal communication, 7 November 2015). In Switzerland and Liechtenstein, the formularies are limited to analgesics, anti-inflammatories, and muscle relaxants. Swiss chiropractors have had these privileges since 1995 [1], and surveys have shown judicious use of this authority in clinical practice [1, 4, 18, 19]. Along with these privileges, chiropractors in Switzerland share primary care status with general physicians in managing patients with spine-related and other MSK complaints [18, 19]. As such, limited prescriptive authority has generally been regarded by Swiss chiropractors as an advantage for the profession in that country [1, 4].

In New Mexico, USA, the chiropractic formulary also contains MSK medications; however, various hormones and other injectable substances for treating non-MSK disorders have been included [17]. Attempts have been made by New Mexico chiropractors to further expand this formulary to include additional prescription drugs and injectable medicines for non-MSK conditions [20]. The motivation behind this push for broader prescriptive authority appears to be that some chiropractors in New Mexico [5] and elsewhere in the United States [21] wish to become ‘primary care physicians.’ There is evidence to suggest, however, that the medical and chiropractic professions would largely oppose such an initiative [6, 8, 11, 20, 22–24], while a collaborative role for chiropractors as ‘primary MSK / spine care providers’ with limited prescriptive authority would be favoured [11, 18, 25–30].

There is also a pressing issue in the chiropractic profession concerning the use of over-the-counter (OTC) medication in clinical practice, which carries both clinical and medico-legal implications. For instance, first line medications suggested for uncomplicated back and neck pain include analgesics, non-steroidal anti-inflammatory drugs (NSAIDs), and/or muscle relaxants [31–38]. As primary contact health care providers for spinal pain and other MSK complaints, chiropractors may be asked by their patients to make recommendations regarding these medications. However, in most jurisdictions, recommending or even giving advice on OTC medications is currently outside of their current legislative scope of practice. Regardless of this ‘barrier,’ there is evidence to suggest that many practising chiropractors continue to make OTC treatment recommendations to their patients [6, 22, 23, 39].

In addition to legislative restrictions, there exists significant contention within the profession as to whether or not medication prescription even belongs in the chiropractic scope of practice [40–42]. Much of this disagreement stems from philosophical differences within the profession [6, 24]. Elements of this discord amongst chiropractors may also be reflective of the number of years in practice, chiropractic college attended, or both [6, 8]. Regardless, with the exception of a few jurisdictions, chiropractors have yet to establish full cultural authority in society under the profession’s current international ‘drug-free’ model [43]. As such, further debate on the issue of prescribing rights in chiropractic is warranted.

The objective of this commentary is to examine the arguments in favour of and against limited medication prescription rights for chiropractors and to discuss the implications of such privileges for the profession. The PubMed, Index to Chiropractic Literature (ICL), and Cumulative Index to Nursing and Allied Health Literature (CINAHL) databases were searched, up to May 1, 2016 using a similar strategy as described previously [40], in order to review the literature for this debate. Additional reference articles that were not identified through the literature searches but were pertinent to this commentary were also included.

## Discussion

### Arguments in favour of limited medication prescription rights for chiropractors

An important first argument in favour of granting limited medication prescription rights to chiropractors is that these privileges would be in line with current evidence-based practice. Currently, most international guidelines recommend, alongside prescription medication, a course of manual therapy and/or exercise as well as education and reassurance as part of a multi-modal approach to managing various spine-related and other MSK conditions [31, 33–38]. Regarding the effectiveness of these and other individual conservative therapies, several studies indicate that no one intervention is clearly superior to another for managing acute MSK disorders [44–46]. As such, if given limited prescription rights chiropractors would gain access to an additional evidence-based modality to effectively manage their patients. This would allow chiropractors to select the most suitable treatment modality, whether pharmacological or non-pharmacological, based on available clinical evidence and patient preference.

There is evidence to suggest that limited medication prescription privileges would also be consistent with chiropractors’ general experience and practice behaviour. For instance, research findings from several studies indicate that regardless of whether or not chiropractors agree with medication prescription rights for the profession, many clinicians tend to recommend OTC medications to their patients in practice [6, 22, 23, 39]. From surveys of chiropractors in Australia [22], the United States [23], and Canada [6], between 66 and 87 % of respondents indicated that they recommend non-prescription analgesics and anti-inflammatories with variable frequency to their patients.

Also in favour of medication prescription is that a more comprehensive treatment approach offered by chiropractors could potentially lead to a reduction in healthcare costs by providing additional specialized health care options for the treatment of MSK conditions. Namely, if patients consult one central practitioner who can effectively address and provide a range of treatment modalities for MSK pain-related matters, the number of visits to providers might be reduced, thereby resulting in better resource allocation. In Switzerland, for example, Houweling et al. [19] found clinically similar pain relief, greater satisfaction levels, and considerably lower overall health care costs in patients who initiated care with a chiropractor versus a medical doctor for the treatment of spinal, hip, and shoulder complaints. In situations where patients present with more complex health problems, chiropractors in primary care could refer such cases to a general physician or specialist for additional examination and/or collaborative management. Such an approach may also result in more streamlined care and reduce waiting times for patients, thus improving the overall quality and delivery of healthcare services.

Limited medication prescription rights could also lead to improved cultural authority for chiropractors and better integration within the healthcare system. Indeed, a strong argument can be made that a profession skilled in the art of manual therapy and possessing limited prescriptive authority would have a significant role to play in the evidence-based spine / MSK care marketplace. Such an advancement has not only the potential of benefiting patients, but also the chiropractic profession as a whole by providing improved professional identity and responsibility. In Switzerland, where the chiropractic profession holds the privilege of limited medication prescription within its scope of practice, chiropractors are fully integrated into the healthcare system and have cultural authority within the MSK domain [18, 19]. Moreover, the large majority of Swiss chiropractors perceive these privileges as an advantage for the profession in that country [1, 4]. In the United States, approximately one-third of all licensed chiropractors currently practising in the state of New Mexico have limited prescriptive authority. This formulary was approved in 2010 [17], and to date there have been no associated injuries or patient complaints registered with the New Mexico Board of Chiropractic Examiners (W. Doggett; personal communication, 31 March 2016).

A final argument in favour of incorporating limited medication prescription into the chiropractic scope of practice is that with these privileges, chiropractors could have a positive influence on public health. For instance, analgesics and NSAIDs are widely used and potentially misused by the general public [47–49], and users are often unaware of the potential side effects that such medication may cause [34, 47]. If granted limited prescriptive authority, chiropractors would be in a position to help advise patients against improper usage of these types of medications. This notion is consistent with current best-practice guidelines [31–38] and appears to resonate with a large number of chiropractors [4, 6, 8, 24, 40, 41]. In two recent survey reports from Ontario, Canada [6, 8], for example, between 68 and 72 % of chiropractic respondents expressed interest in gaining limited prescription privileges because of this potential role for the profession.

### Arguments against limited medication prescription rights for chiropractors

Although limited medication prescribing rights for chiropractors may seem appealing to many, there are a number of hurdles to overcome including political and legislative challenges. These will not likely be easy to surpass and may require much time and a united effort on the part of the profession. In the United Kingdom, for example, the physiotherapy profession campaigned for nearly a decade before being granted limited independent prescribing rights by the government in that country [12]. For chiropractors, there will be major changes needed before prescription rights can be incorporated into the profession’s scope of practice. Chiropractors and their governing bodies who wish to pursue limited prescriptive rights for the chiropractic profession should start reaching out to politicians and third-party payers to promote the benefits of making such changes to the existing healthcare system [26, 50]. Additional research may be needed to better understand the consequences of such changes and provide leverage for discussions with healthcare stakeholders.

Existing healthcare legislation needs to be amended in order to regulate medication prescription by chiropractors. Both national and regional levels must be involved so that patients and pharmacists know which medications are approved for prescribing by chiropractors and which medications are covered by insurance policies. Moreover, checklists should be designed to provide guidance on safe medication prescription for different clinical scenarios. These procedures may also assist chiropractors in deciding when to communicate with other health care practitioners so as to avoid multiple prescriptions for the same patient complaint.

Notwithstanding the importance of such changes, there is a need to focus on the curriculum of chiropractors. Inadequate knowledge and competence can result in harm to patients; therefore, appropriate and robust continuing education and training would be an absolute requirement [51]. At present, most chiropractic students internationally receive an average of only 12 h of coursework in pharmacology and toxicology [52]. In contrast, chiropractic students in Switzerland receive over 80 h in pharmacology and toxicology at the University of Zürich [6]. Clearly, there exists a need to revamp the undergraduate pharmacology curriculum of non-Swiss chiropractic colleges if the profession wishes to lobby for scope expansion including limited prescriptive authority. Emphasis should not only be placed on didactic education of prescribers, but also on practical supervision and training by experienced tutors. This could be performed by implementing a mandatory postgraduate program for chiropractors including supervised experience in a clinical setting. Such a program has been successfully implemented in Switzerland. In addition to working under the supervision of an experienced chiropractor for a minimum of two years, new chiropractic graduates are required to perform clinical rotations at specialized hospital wards including orthopedics, rheumatology, and physical medicine [53, 54]. Chiropractors who wish to practise in Liechtenstein must also complete this same postgraduate training. In New Mexico, USA, chiropractors must complete a two-year postgraduate Master of Science degree in ‘Advanced Clinical Practice’ [26, 55] before they can obtain a license to prescribe from the limited chiropractic formulary in that state [17]. A similar academic program is now being proposed for chiropractors seeking legislative scope of practice expansion with limited prescriptive authority in the state of Wisconsin [26, 30].

Another important issue to consider regarding chiropractic prescribing rights relates to the divisiveness around this topic within the profession. In fact, some have argued that the right to prescribe medication in chiropractic practice is the profession’s most divisive issue [41]. While it may be true that chiropractors have generally been split in their opinions concerning the use of prescription medications in chiropractic practice [40–42], there is evidence to suggest that this division relates to differing philosophical ideologies within the profession and that those who are against gaining prescription privileges for chiropractors are in the minority. For example, in surveys [6, 24] of chiropractors in the United States, Canada, and Mexico, the large majority of respondents who classified themselves as practising within a “broad” (or ‘mixer’) scope of chiropractic practice were in favour of the idea of gaining prescriptive rights while the large majority of those who identified themselves as “focused scope” (or ‘straight’) chiropractors were opposed. Broad scope chiropractors made up approximately one-third of the respondents in these studies compared to focused scope chiropractors who made up less than two-fifths. Of the remaining and largest group of “middle scope” respondents, the majority also favoured chiropractic prescribing rights [6, 24]. This indicates that there may be potential for consensus among the majority of chiropractors regarding medication prescription rights for the profession. Interestingly, focused scope respondents were not unanimously opposed to the notion of chiropractic prescribing rights in these studies. In fact, between 18 and 24 % of focused scope chiropractors indicated at least some level of support for limited (i.e. MSK) medication prescription privileges [6, 24]. These findings demonstrate that there is potential for unity on this topic within the profession.

Irrespective of philosophical differences, there is evidence to suggest that there could be a shift in chiropractors’ attitudes toward medication prescription rights occurring within the profession in general. In particular, results from a growing number of surveys indicate that chiropractic clinicians who are interested in prescription rights seem to be most in favour of gaining an expanded scope to allow for the limited prescription of medications for common MSK conditions. In various surveys of chiropractors practising in Australia, the United States, Canada, Mexico, and the United Kingdom, majorities of between 54 and 78 % of respondents expressed interest in the expansion of scope of practice, including the addition of prescriptive authority for a select number of medications such as NSAIDs, analgesics, and muscle relaxants [2, 3, 6–8, 22–24] (2007 and 2011 Ontario Chiropractic Association member surveys: B. Haig; personal communication, 3 November 2014). Various chiropractors and chiropractic associations have also published a number of recent policy statements, commentaries, and policy papers [25, 26, 50, 56, 57] calling for similar legislative changes to the chiropractic scope of practice, reiterating that the profession may not be as divided on this topic as previously thought [40–42]. Owing to the fact that several of the aforementioned surveys were unpublished [2, 3, 7] (2007 and 2011 Ontario Chiropractic Association member surveys: B. Haig; personal communication, 3 November 2014) or were limited by low response rates [6, 8, 22–24], these findings should be interpreted carefully.

An issue often raised concerning chiropractic prescribing rights relates to the influence these privileges could have on the profession’s identity. For instance, throughout most of its 120-year history the chiropractic profession has presented itself as a drugless, non-surgical healing profession [40]. Some have argued that further incorporation of prescription rights into the chiropractic scope of practice will negatively impact the distinct professional brand and identity of chiropractic [41]. Others have argued however that chiropractic has always lacked a clear professional identity, resulting in its failure to establish full cultural authority and respect within mainstream society [25, 57–59].

Aside from ideological and philosophical barriers, a further argument against introducing limited medication prescription rights into the chiropractic scope of practice is that such privileges would increase chiropractors’ professional responsibilities. For example, if given limited prescriptive authority, chiropractors would be required to recognize and monitor medication side effects in their patients [60]. Additional implications would include necessary increases to a practitioner’s liability insurance coverage as well as potential conflict of interest/ethical issues related to dispensing and/or selling prescribed medications [11]. For instance, it is well-documented that prescribing practices can be influenced through participating in continuing education programs funded by pharmaceutical companies [61, 62]. These additional requirements and ethical concerns might constitute drawbacks for a number of chiropractic clinicians.

Prior to medication prescription rights being incorporated into the chiropractic scope of practice worldwide, further discussions need to take place around the breadth of such privileges for the chiropractic profession. For instance, although there may be a growing interest among chiropractors internationally towards gaining limited prescriptive privileges for treating MSK conditions, there does not seem to be the same level of support within the profession concerning that of full prescribing rights. From results of several published surveys [6, 8, 22–24] of chiropractors in Australia, the United States, North America, and Canada, respondents have generally been opposed to the idea of chiropractors prescribing medicines for non-MSK conditions. Practising chiropractors have also indicated that their knowledge of these types of medications is insufficient [6]. Some chiropractors and educational institutions in the United States nevertheless appear to be lobbying for expanded scope of practice legislation in order for chiropractors to become primary care physicians with full prescriptive authority [20, 21]. However, research suggests that only a small minority of chiropractors in clinical practice would actually be interested in a plenary scope [6, 24]. For example, in their survey, Emary and Stuber [6] found only 11.6 % (111/960) of respondents agreed that chiropractors should be able to gain an expanded scope of practice to allow for the prescription of any and all medications, including controlled substances. Similar results were found by McDonald et al. [24] in their survey of practising chiropractors from North America. One possible solution to this problem would be for chiropractic regulatory boards to limit medication prescription privileges to analgesics, NSAIDS, and muscle relaxants, such as is the case for chiropractors in Switzerland and Liechtenstein, so as to focus on treatment modalities that are compatible with chiropractic practice.

## Conclusions

The right to prescribe medication in chiropractic practice may not be as divisive an issue within the profession as previously thought. In particular, there appears to be a growing interest among chiropractic clinicians towards gaining prescription privileges for treating spine-related and other MSK conditions. If limited to such a scope, prescription rights for chiropractors would be in line with current evidence-based practice, common chiropractic practice behaviour, and interdisciplinary collaboration with medical doctors. Moreover, limited chiropractic prescribing rights could result in significant health cost savings, strengthening of the chiropractic profession, and an overall positive influence on public health.

Although prescription rights may seem appealing to many in the chiropractic profession, major changes to education and legislation will first be needed before these privileges can be incorporated into the profession’s scope of practice. Chiropractors and the governing bodies that wish to pursue limited prescriptive authority should engage in dialogue with like-minded politicians and third-party payers and promote the benefits of making such changes to the existing healthcare system. In addition, future research might be needed in order to justify such discussions with healthcare stakeholders.

## Abbreviations

CINAHL, cumulative index to nursing and allied health literature; ICL, index to chiropractic literature; MSK, musculoskeletal; NSAIDs, non-steroidal anti-inflammatory drugs; OTC, over-the-counter; USA, United States of America
